# Delirium

**Published:** 1904-10

**Authors:** 


### DELIRIUM.*

Delirium is a symptom of intoxication and not of insanity.

This assertion I believe to be in accordance with fact; the exceptional conditions, in which this mental manifestation is not caused by some sort of poison, are so few that they "prove the rule."

There are two kinds of toxemia which may produce delirium, that following the use of certain drugs and that induced by sepsis.

### DRUG POISONING.

Alcohol used as a beverage is followed by intoxication. The boy or man who has, for the first time, taken a very moderate quantity

*A paper read by Dr. Gershom H. Hill at the Annual Meeting of the Austin Flint-Cedar Valley Medical Society, Waterloo, July 13.

of champagne, or other wine or of beer, is likely not only to talk silly, but also in an incoherent manner, to himself or to some imaginary person. Reproof, sharply spoken, or a hard slap on the shoulder, may temporarily relieve this slight delirium. On the other hand, the inebriate may become a "raving maniac;" he has suddenly, or by degrees, poisoned himself so completely that he is in danger of death; he sees the devil, fears him, tries to escape from him; he can not eat nor sleep; he is not insane, but is a case of delirium tremens.

Besides alcohol, in its various forms, ether, chloroform, opium, belladonna, and a few other drugs may poison the system to such an extent as to cause delirium.

### SEPTIC POISONING.

In recent years the attention of the profession has been called to a condition of the blood and of the system known as auto-intoxication. We have learned not only that the nervous system is thus disturbed, but that a "flighty" condition of the mind may thus be produced.

The earliest, the simplest and the most transient form of delirium caused in this manner, is observed in little children, who suddenly develop an elevation of body temperature, due to catching cold, to indigestion or to constipation. The parent hears the child, who is healthy and strong, talking in his sleep, quietly to himself, or in a loud and excited manner he is scolding some one else.

Night terrors sometimes occur in children aged between three and eight years. Such cases are either idiopathic or symptomatic in character. Nervous and imaginative children are prone to this kind of excitement. They are timid, shy or self-conscious, intense in feeling or in conduct. When analyzed carefully, this mental condition is quite different from delirium.

Acute infectious diseases are sometimes attended by delirium. In severe cases of typhoid fever delirium is present. Usually it does not develop until the second and sometimes not until the third week. It may be slight and only nocturnal. It is, as a rule, a quiet delirium, though there are cases in which the patient tries to get out of bed and escape from his room. Pneumonia is an infective disease, caused by a diplococcus, which has its seat, for the most part, in, and produces its chief effect upon, the lung; it may, however, invade the meninges. Delirium is seldom manifested before the third stage of pneumonia, when the blood is most heavily loaded with septic material.

### PUERPERAL INSANITY.

Puerperal insanity is so called if the mental derangement developes within six weeks of child-birth. The most common cause of it is predisposition from heredity, or some constitutional lack of stability. However, the exciting causes, which co-operate to dethrone the reason, at this particular time, are mental strain from anxiety, loss of sleep, or from pain. This condition may be manifested by either excitement or despondency.

Again, excessive loss of blood may be followed by symptoms of melancholia, which in time is remedied by proper nutrition and good care. The third puerperal condition is septic and manifested by delirium. The patient may suddenly become wild and restless, altogether unnatural. The mental condition is acute, it is due to poisoned blood circulating in the brain. Perhaps the placenta has not been entirely removed, or absorption may have taken place from a lacerated cervix, or from some other wound. Occasionally the vitiated condition of the blood is the consequence of uncleanliness. Since the exciting causes of puerperal mania can be removed, most cases recover at home in a short time.

### ACUTE DELIRIOUS MANIA.

In 1849, Luther Bell described a form of insanity which he called typho-mania. Cases of this kind are cited by many of the old writers. The disease is characterized by maniacal excitement which is violent and continuous. The patient can not sleep, will not eat, is very restless, is suspicious of all who undertake to care for him, will pay attention to nothing and is distracted by hallucinations which he can not explain. The pulse is frequent, small and irregular, temperature several degrees above normal, the tongue is dry, rough and may be black, sordes on lips and teeth, thirst and obstinate constipation. The eyes are dry, sunken and unnatural. The disease may be preceded by a period of irritability, restlessness and insomnia, or more often the symptoms develop suddenly. Very acute cases may terminate within a week; others may continue two or three weeks, most all of them succumb to exhaustion.

Osler in his book on Practice declares that the nature of this disease is unknown, but says that some of the cases suggest acute infection. In my opinion, this is the probable explanation for all the cases.

With prompt and skillful attention, if this disease is not prevented, it is relieved so that now acute delirious mania furnishes only a very small percentage of the patients admitted to the State hospitals.

### OTHER DELIRIOUS CONDITIONS.

In acute meningitis, the mind may wander, the inflammation may be accompanied by sepsis. Fractures of the skull and other injuries which affect the brain are likely to produce mental disturbances of various kinds, but they are not insanity and are quite easily differentiated from it.

"Muttering Delirium," in exhausted patients, is a symptom of impending dissolution, due to an insufficient blood supply to the brain or to a depraved condition of this vital fluid. This sort of wandering of the mind develops gradually and is passive in character.

### DEFINITIONS AND CONCLUSIONS.

Delirium is an acute, transitory change in the mind of an individual, it is usually an excited condition and accompanied by an elevation in body temperature. Etymologically, it means to wander from the furrow. It is characterized by restlessness, incoherence of speech, imaginations and sensory perversions. Insanity, on the other hand, is a chronic state of mind, without much constitutional disturbance; it is a disease of the brain, in which gross lesions are absent, pathological changes are seldom visible to the unaided eye, but it causes a prolonged departure of the mind from its usual mode of operation. Insanity is measured by months and years, not by days.

If delirium is considered as an entity, emanating from a poisoned brain, or from one that is acutely inflamed, it can almost invariably be distinguished from the delusions, the hallucinations, and the incoherent speech which are symptoms of insanity.—*Iowa Medical Journal.*

